# Annus Medicus, 1894

**Published:** 1895-01-05

**Authors:** 


### The Hospital

An Institutional Journal of

The Medical Sciences and Hospital Administration, with

Vol. XVII.—No. 432.] AN EXTRA NURSING SUPPLEMENT. [Jan. 5, 1895.

### Annus Medicus 1894.

### A Medical Weakness.

One of the weakest of not a few weaknesses in modern medicine is its lack of critical power. Everybody struggles in the arena, everybody strives for pre-eminence and notoriety, but nobody sits on the judgment seat. What is still worse, the judgment seat itself is discredited. There are in medicine, particularly in British medicine, many critics of the most experienced and judicial order; but nobody wishes to hear a word they have got to say. Not unnaturally, therefore, they preserve silence. But if a man does but start a new theory of treatment, or revive an old operation, immediately the whole of European and American medicine rushes to his feet to acclaim him an eighth wonder of the world. Concerning which the most appropriate criticism that can be offered is perhaps that which concludes a good many hypothetical propositions in Geometry, *Quid est absurdum.* In our brief summarisation of the work of the *annus medicus* 1894 we do not propose to fall into the contemptible and almost criminal weakness of indiscriminate laudation. Our remarks will be at least conceived in a critical spirit, though to profess oneself a medical critic at the present time is almost to invite contempt. Nevertheless, in our judgment, competent criticism is one of the greatest services which can be rendered to the whole science and art of medicine of the day.

### Serum Therapy.

The medical sensation of the year has undoubtedly been the application of serum therapy in the treatment of diphtheria. Who deserves the credit of this? Whether Roux, or Behring, or another, is a question not worthy of discussion, at any rate until certain other questions of real importance are judicially and finally decided upon. There is no *à priori* unlikelihood that animal serum will prove itself a powerful therapeutic agent. On the contrary, the probabilities are all in its favour. If certain active principles found in plants shew therapeutic properties when administered to human beings and the lower vertebrates, much more, one would think, is it to be expected that what may be called "animal active principles" will be potent in their effects when administered to animals. Animal serum has had its fascinations for physiological investigators ever since physiological investigation began to be scientifically practised. Long before Roux or Behring announced its wonderful potency in diphtheria Arthur Klein had pointed out its probable efficacy in some such terms as these: "The study of blood serum may affect the whole future of medicine. Immunity, which is the power of an organ to defend itself successfully against the entrance of infection, is either congenital, and peculiar to the species, or acquired. Two explanations are suggested for acquired immunity. One supposes that the white blood corpuscles take up and destroy the cause of infection; the other that the agency of protection consists in substances dissolved in the blood, that is in anti-toxines. It is believed that the serum of immune animals contains these anti-toxines."

Obviously we have here a theory which covers the whole ground of preventive medicine. Is it not imperative, therefore, that every experimental step shall be taken with the most scrupulous care, that every fact shall be noted with the most rigid accuracy, that every induction shall be of the amplest magnitude, and that every conclusion shall be demonstrated by such an array of facts and so complete a chain of logical reasoning that it can by no possibility be successfully assailed? Is it not plain that in such an investigation as this the practical success of medicine as an art, and its honour as one of the demonstrable sciences, are alike at stake? Let then such an investigation be pursued with all that absence of unscientific frivolity, all that sobriety of judgment, all that regard for absolute truth, and all that broad intellectual competence which the nature of the case so imperatively demands.

Serum therapy has not been employed in the therapeutics of diphtheria alone. Andre has published in detail two cases of small-pox treated with hypodermic injections of serum obtained from the blood of patients who had previously suffered from the disease. So far as he was able to discover no modifications of any kind in the disease resulted from the treatment. If Andre had treated two hundred instead of only two cases in this way, and with like negative results, it is obvious that a fact of the most important bearing on the whole question of serum therapy would have been established. It will be remembered also, in this connection that mallein has been used for the immunisation of horses against glanders. The serum of horses so immunised has been employed in the treatment of glanders; and the controversy as to its value as a remedial agent is still tearing the veterinary world to pieces.

The Anti-toxin Treatment of Diphtheria.

If those who desire to form correct judgments on such an important question as that of the anti-toxin treatment of diphtheria will approach the subject with such facts and considerations in their minds as we have now indicated, they will be enabled to arrive at rational conclusions, and not be driven like flocks of sheep to follow the bell-wether whithersoever she may happen to lead. Behring has employed anti-toxic serum in some hundreds of cases of diphtheria at Berlin, and he announces a mortality diminished by one-half. Roux has also treated in Paris a number of cases amounting to hundreds, and he announces similar or even more favourable results. The treatment has spread throughout France with the rapidity of wildfire—a grim commentary upon all the methods of treatment which have preceded it. At Berlin the question has formed the subject of a full-dress discussion by the faculty, and the usual scenes have been witnessed. Almost everybody professed himself an anti-toxinist, and, as a result, was cheered to the echo. Hansemann, an admittedly competent critic, took an opposite view, and aroused the astonished indignation of thousands of doctors throughout the civilised world, doctors whose knowledge of throat affections is absolutely infinitesimal compared with that of the distinguished German specialist. It is true that Virchow, compelled, as he affirmed, "by the brute force of figures," gave in his adhesion, for the time at any rate, to the treatment. And so, indeed, do we all. We cannot do otherwise. "The brute force of figures" makes it compulsory upon us at least to try the treatment, and to try it with the utmost thoroughness. But that "brute force" does not compel us at the same time to abdicate our reason, and to fling away our independence of mind. On the contrary, it impels us to try the new remedy in a serious, careful, and critical spirit; to try it in a sufficient number of cases; and to make known the results of our testing with as much confidence and courage as if the final decision for the whole world rested entirely upon ourselves. If medical men would but act in this way instead of following prominent names and despising each other, the medicine of Europe and America as a whole would soon occupy a very different position from that which belongs to it to-day.

Anti cholera Inoculations.

On the same lines of investigation as we have already discussed are the anti-cholera inoculations which are being carried out in India by M. Haffkine under the auspices of the Indian Government. This is a subject which every medical man who possesses any scientific instincts at all, or who has merely an ordinary regard for the honour and progress of his profession, must have watched with a deep and constantly growing interest. Announcements of glowing success have been followed by reports of overwhelming failure; and these again have been succeeded by assurances of at least partial success. In Dr. Simpson, of Calcutta, the medical profession recognises a judicious and honest critic. On the one hand he is inspired by an ardent zeal for the progress and elevation of his profession; on the other, his mind is guided and controlled by the reasonableness born of experience and an innate love of truth. With a critic like Dr. Simpson close at hand M. Haffkine will be under no temptation to magnify those facts which are in his favour, or to minimise those which make against him. Moreover, he will carry on his work with the confidence which arises from the assurance that that work is being judged and reported upon by an impartial critic who has the respect of all intelligent medical men in both hemispheres.

Nothing could more convincingly show the necessity for a critical attitude of the general medical mind than these very investigations in anti-cholera inoculation which are now being carried out in our great Eastern dependency. Those who committed themselves at the outset to an uncompromising belief in the efficacy of the inoculations have had the edifice of their confidence rudely tumbled about their ears. Those, on the other hand, who, on the strength of one apparent failure, were jubilant over the announcement of a temporary ill-success, will now feel that there would have been wisdom in waiting for evidence of a more convincing kind. The judicious, who neither hungered too greedily for success, nor triumphed over premature evidences of failure, are having, as is not unusual, decidedly the best of it. The conclusion, of course, is obviously this, that the subject of anti-cholera inoculations is both great and difficult; that it demands investigation under a vast variety of conditions; that it must be dealt with from many standpoints, in many different situations and circumstances, and by a sufficient number of scientifically-trained persons of varying and quite independent mental characteristics. It is, in short, one of those provinces of scientific advancement of which medical investigators, as yet, have touched but the outermost fringe.

Bacillary Immunisation against Erysipelas.

Still investigating the field of bacillary medicine, Dr. W. S. Melsome and Mr. Louis Cobbet have published important results in immunisation. The field chosen was that of erysipelas, and the experiments were made with cultivations of streptococcus erysipelatis. Results of the profoundest significance, as will be seen by the philosophic medical inquirer, were obtained. It was found, for example, that local inoculations produced local immunisations; that inoculations made ventrally, or, in other words, into regions of more vital significance, produced immunisation which tended to be more general in character. But it was also shown, and these are facts of very great scientific significance, that the immunisations, whether local or general, were of exceedingly brief duration, lasting, in the longest instances, for months only, in most cases only for weeks, and in many merely for days. It is obvious then that so far as the bacillary disease of erysipelas is concerned, inoculations will be quite futile unless we are prepared to inoculate every few weeks or months.

### More Light upon Vaccination.

Important light, it seems to us, is thus thrown upon the burning question of vaccination against small-pox. If local inoculation with streptococcus erysipelatis produces results which are both local and temporary, is it not possible that the results of local inoculation with vaccine lymph may be both more local and more temporary than some people may have hitherto been inclined to believe? And is it not conceivable, also, that inoculations with vaccine lymph made into parts of more vital significance than the forearm, the upper arm, or the leg, might produce results which would be both more general and more permanent in their effects than any that are produced by our present methods of vaccination? One thing at any rate is clear, that all these questions of the absolutely scientific prevention of disease are but in their scientific infancy. Much knowledge of a highly serviceable kind has been gathered by experience and what may be called "rule of thumb" in every part of the world. But what we desire now, and never can be content until we have conquered, is the inner secret, the *modus operandi*, and the precise limits, both as to duration and extent, of all such methods of treatment as vaccination and inoculations in general. Here is a field which in all probability will not have been completely investigated when the youngest and least experienced of our present year's medical freshmen shall have been laid to rest in his grave.

### A New Air Cure for Tuberculosis.

Before passing from what may be called the "higher peaks" of medical progress to the more familiar levels of common practice we may briefly notice a fresh attempt to deal in a practical way with tuberculosis. If the method, as is now hoped, should prove in any high degree successful with children, it will very speedily be extended to all classes of cases. The system, which after all is novel rather in its methods than in its principles, consists in the combined use of air and a variety of bactericides. A kind of school hospital has been opened at Ormesson, close to Villiers, on the river Marne. One portion of the building is devoted exclusively to the treating of tuberculous children, and is separated by the whole length of the garden from the main structure. On the ground floor are the consulting and operation rooms, inhalation and bath rooms, and a bacteriological laboratory. The wards are supplied with ozone from leather bags. Sun and air baths are taken on a terrace furnished with comfortable couches for the purpose. Kerosene, turpentine, eucalyptus, and similar bactericides are mixed with the atmospheric air of certain portions of the building. There are no published results yet available, but the experiment, though by no means so sensational as some of those to which we have been recently accustomed, is of a decidedly interesting character.

### Internal Surgery.

The surgeon, it is often said by the unphilosophical, is rapidly pushing the physician into the remoter fastnesses of the human organism, and may some day, it is thought, bow the learned gentleman out of the higher regions of practice altogether. This is a view which only the ignorant can hold. In ultimate analysis there can be no divorce between medicine and surgery. They are not two, but one; and not one by marriage union, but by natural genesis and birth. The surgeon is pushing his way into internal cavities, into the chest, into the abdomen, everywhere. But what is it which has enabled him to be so bold with the knife, the exploring finger, and the probe? It is a medical, or if we must be hyper-scientifically precise, a chemico-medical discovery which has opened to him the gateways of these internal cavities and endowed him with the power of moving about safely and freely therein. It is the discovery of medical and surgical antisepticism. Dr. Arnold Chaplin, in a paper published in the *Practitioner*, discussed fully the wisdom or unwisdom of opening and draining tuberculous pulmonary cavities. Up to the present time, notwithstanding Dr. Pendleton Dandridge's enthusiastic advocacy, it cannot be said that the surgical opening of definitely tuberculous cavities in the lungs is an operation which is more than occasionally justifiable. The physiological reasons which render such an operation both dangerous and uncertain are too obvious to call for discussion here.

Mr. John Malcolm has published an interesting case of nephrectomy carried out at the Samaritan Hospital in 1893. Sir Spencer Wells and Mr. Knowsley Thornton acted in concert with Mr. Malcolm at the earlier consultations. The case was one of a child two years of age with a large malignant growth adherent to the right kidney. The point of interest to all practitioners is that notwithstanding the malignant nature of the growth the child is now well, sixteen months after the date of removal. What may be the ultimate history of the case it is impossible even to guess, except, indeed, that if past experience is to be taken as our guide the chances are as more than ten to one against permanent restoration to health.

### The Growth of Knowledge in Lunacy.

This journal may claim some credit for the publication during the past year of an important series of articles on "Lunacy" by Dr. T. S. Clonston, the distinguished superintendent of the Morningside Lunatic Asylum at Edinburgh. He has brought to a focus the assured knowledge which alienists have long been accumulating. Chiefly, he has re-stated the established medical conviction that insanity is always and everywhere a physical disease, and not an aberration in morals or will. Starting from this basis, he has insisted that lunatics are patients, not prisoners, and that lunatic asylums are not prisons, but hospitals. Carrying out these ideas into social and legal relations, he has contended that the responsibility of the lunatic is so varying and peculiar a quantity that no person not a trained expert can form any adequate judgment of its nature and value. He has affirmed the position so uniformly taken up by all competent authorities on the subject, that the lunatic from first to last is to be placed under medical charge, and that medicine alone is to decide his fate in every relation of life. This is the philosophical and the righteous position; and to this position all statesmen and lawyers, as well as all other men, will finally come, if civilisation continues to advance on the lines of sound science and sense.

### General Practice.

Of all the departments of medical activity, that which is known as "general practice" is the most extensive and the most important. Whilst it is true that the specialist has rendered services to medicine which can never be forgotten, and has, indeed, been the maker of modern medicine as a science and an art, it is equally certain that he has been but the pioneer and conqueror of new tracts of country which the general physician and surgeon will slowly enter and finally occupy altogether. Fifty years ago the progress of medicine imperatively demanded Specialism. To-day the progress of general medical education is handing over the specialist's knowledge to the generalist. This is entirely as it should be; it is the consummation which every medical philosopher is everywhere devoutly wishing for.

Great is our satisfaction, then, to note in the ranks of general practice, among family physicians as well as among general physicians and surgeons of consultant status, a growing culture, an eager strenuousness of mind, a diligent study of principles, and a boldness in their clinical application, which give promise of steady and continuous progress in every department of medical life. By way of illustration we may instance the rapidity with which cold and tepid bathing as a part of the treatment of typhoid and other fevers has risen into favour among practitioners of every class. Undoubtedly the most certain and the most rational method we know of lowering temperature is the intelligent use of such a cooling agent as water. But we were not prepared to hear, as we did the other day from an enthusiastic layman, that a newly-graduated medical assistant in a remote country district had boldly stepped in with the cold water treatment and cured a patient of typhoid fever when two or three of the older practitioners in the neighbourhood had affirmed their conviction that he must certainly die. Many other illustrations of the wide spread of knowledge and the improvement of technique might be cited from all parts of our own country and of Europe and America, did space permit. Right glad and thankful must every physician be who holds himself but as one member of a vast army whose great purpose it is to annihilate physical ills and to make mankind the possessor of a happy because a healthy world.

### Obituary.

We close this brief sketch of a year's ardent medical activity with an obituary notice of a few of the leading names in medicine which have disappeared from our ranks during the past year. Let us give the place of honour to Oliver Wendell Holmes, not for that he was the most scientific, but because he was the most human of them all. Helmholtz, too, is dead, and Professor Milnes Marshall, of Manchester, the latter in the flower of his age; Browne-Séquard is also dead; and Dr. Octavious Sturges and Professor Leishman, of Glasgow. The oldest medical graduate of the University of Oxford, Dr. Francis Bissett Hawkins, has died at the age of 98. Inspector-General Robert Lawson, Honorary Physician to the Queen; Mr. Henry Smith, of King's College; Miss Jessie Flewitt Hatch, M.B., one of the resident medical officers of the North-Eastern Fever Hospital; Mr. John Clay, of Birmingham; Dr. William Bell Hunter, and Dr. Withers Moore, with many others, have passed from the ranks of the living. The year has been one of great mortality among great medical names. Let our sorrowful reverence go with the dead. The living who are attracted to medicine by its science and the largeness of its humanity are an increasing and an increasingly intellectual host. Never did our great profession, more than at the present moment, give promise of a progress at once so noble, true, and deep. Of all the sciences medicine is the most human, and should command from every human being an ardent aspiration for its advancement towards a constantly receding but always striven-for goal of scientific perfection.

